# Accuracy and usability of a diagnostic decision support system in the diagnosis of three representative rheumatic diseases: a randomized controlled trial among medical students

**DOI:** 10.1186/s13075-021-02616-6

**Published:** 2021-09-06

**Authors:** Johannes Knitza, Koray Tascilar, Eva Gruber, Hannah Kaletta, Melanie Hagen, Anna-Maria Liphardt, Hannah Schenker, Martin Krusche, Jochen Wacker, Arnd Kleyer, David Simon, Nicolas Vuillerme, Georg Schett, Axel J. Hueber

**Affiliations:** 1 Department of Internal Medicine 3 - Rheumatology and Immunology, Friedrich-Alexander University (FAU) Erlangen-Nürnberg and Universitätsklinikum Erlangen, Ulmenweg 18, 91054 Erlangen, Germany; 2 Deutsches Zentrum für Immuntherapie (DZI), Friedrich-Alexander-University Erlangen-Nürnberg and Universitätsklinikum Erlangen, Erlangen, Germany; 3 AGEIS, Université Grenoble Alpes, Grenoble, France; 4 Medical Department, Division of Rheumatology and Clinical Immunology, Charité-Universitätsmedizin Berlin, Berlin, Germany; 5 Institut Universitaire de France, Paris, France; 6 LabCom Telecom4Health, University of Grenoble Alpes & Orange Labs, Grenoble, France

**Keywords:** Clinical decision support system, Diagnosis, eHealth, Accuracy, Apps

## Abstract

**Background:**

An increasing number of diagnostic decision support systems (DDSS) exist to support patients and physicians in establishing the correct diagnosis as early as possible. However, little evidence exists that supports the effectiveness of these DDSS. The objectives were to compare the diagnostic accuracy of medical students, with and without the use of a DDSS, and the diagnostic accuracy of the DDSS system itself, regarding the typical rheumatic diseases and to analyze the user experience.

**Methods:**

A total of 102 medical students were openly recruited from a university hospital and randomized (unblinded) to a control group (CG) and an intervention group (IG) that used a DDSS (Ada – Your Health Guide) to create an ordered diagnostic hypotheses list for three rheumatic case vignettes. Diagnostic accuracy, measured as the presence of the correct diagnosis first or at all on the hypothesis list, was the main outcome measure and evaluated for CG, IG, and DDSS.

**Results:**

The correct diagnosis was ranked first (or was present at all) in CG, IG, and DDSS in 37% (40%), 47% (55%), and 29% (43%) for the first case; 87% (94%), 84% (100%), and 51% (98%) in the second case; and 35% (59%), 20% (51%), and 4% (51%) in the third case, respectively. No significant benefit of using the DDDS could be observed. In a substantial number of situations, the mean probabilities reported by the DDSS for incorrect diagnoses were actually higher than for correct diagnoses, and students accepted false DDSS diagnostic suggestions. DDSS symptom entry greatly varied and was often incomplete or false. No significant correlation between the number of symptoms extracted and diagnostic accuracy was seen. It took on average 7 min longer to solve a case using the DDSS. In IG, 61% of students compared to 90% in CG stated that they could imagine using the DDSS in their future clinical work life.

**Conclusions:**

The diagnostic accuracy of medical students was superior to the DDSS, and its usage did not significantly improve students’ diagnostic accuracy. DDSS usage was time-consuming and may be misleading due to prompting wrong diagnoses and probabilities.

**Trial registration:**

DRKS.de, DRKS00024433. Retrospectively registered on February 5, 2021.

**Supplementary Information:**

The online version contains supplementary material available at 10.1186/s13075-021-02616-6.

## Introduction

Rheumatology encompasses various rare diseases, and it often takes months to establish the correct diagnosis [1]. The typical symptoms are often common, unspecific, and difficult to evaluate for patients and health care providers [2, 3]. General practitioners (GP) often have trouble identifying inflammatory rheumatic musculoskeletal diseases (RMD) correctly [4] and only based on patient medical history and clinical examination; even experienced rheumatologists correctly identified only 27% of inflammatory RMD [5].

The majority of mistakes in the diagnosis are caused by cognitive errors [6], and besides educational strategies, checklists have successfully been implemented in medicine to reduce errors and increase treatment quality [7], e.g., reducing central line infection rates [8] and surgical complications [9]. Diagnostic decision support systems (DDSS) and symptom checkers (SCs) are increasingly used [10–12] and promise to help patients and healthcare professionals to establish the correct diagnosis by providing a checklist of differential diagnoses [13]. Previous studies showed that the majority of general practitioners found using a DDSS useful [11], using DDSS had no negative effect on patient satisfaction [11], and showed potential to increase diagnostic accuracy [14, 15] without increasing consultation time [14]. Furthermore, DDSS usage can significantly increase physician’s certainty about a diagnosis [14]. Currently, more than 100 SCs exist [16], and SCs like Ada have been consulted more than 15 million times in 130 countries [17]. Ada is a Conformité Européenne (CE)–certified medical app that is freely available in multiple languages [17]. Previous studies reported favorable results for the Ada symptom checker, suggesting that rare diseases could be diagnosed earlier [18, 19], mental disorders could efficiently be screened [20], and Ada have the best overall diagnostic accuracy and condition coverage [21]. The need for evaluation of decision support systems for the diagnosis of rheumatic diseases has been postulated already in 1991 [22]; however, despite greatly improved technology, evidence still lags behind. We hypothesized that inexperienced future physicians might benefit from using a DDSS to solve rheumatic cases.

The aim of this study therefore was to evaluate the impact of Ada, an artificial intelligence (AI)–assisted DDSS in the diagnosis of rheumatic diseases in a manufacturer-independent randomized controlled trial.

## Methods

### Intervention and randomization

**Fig. 1:**
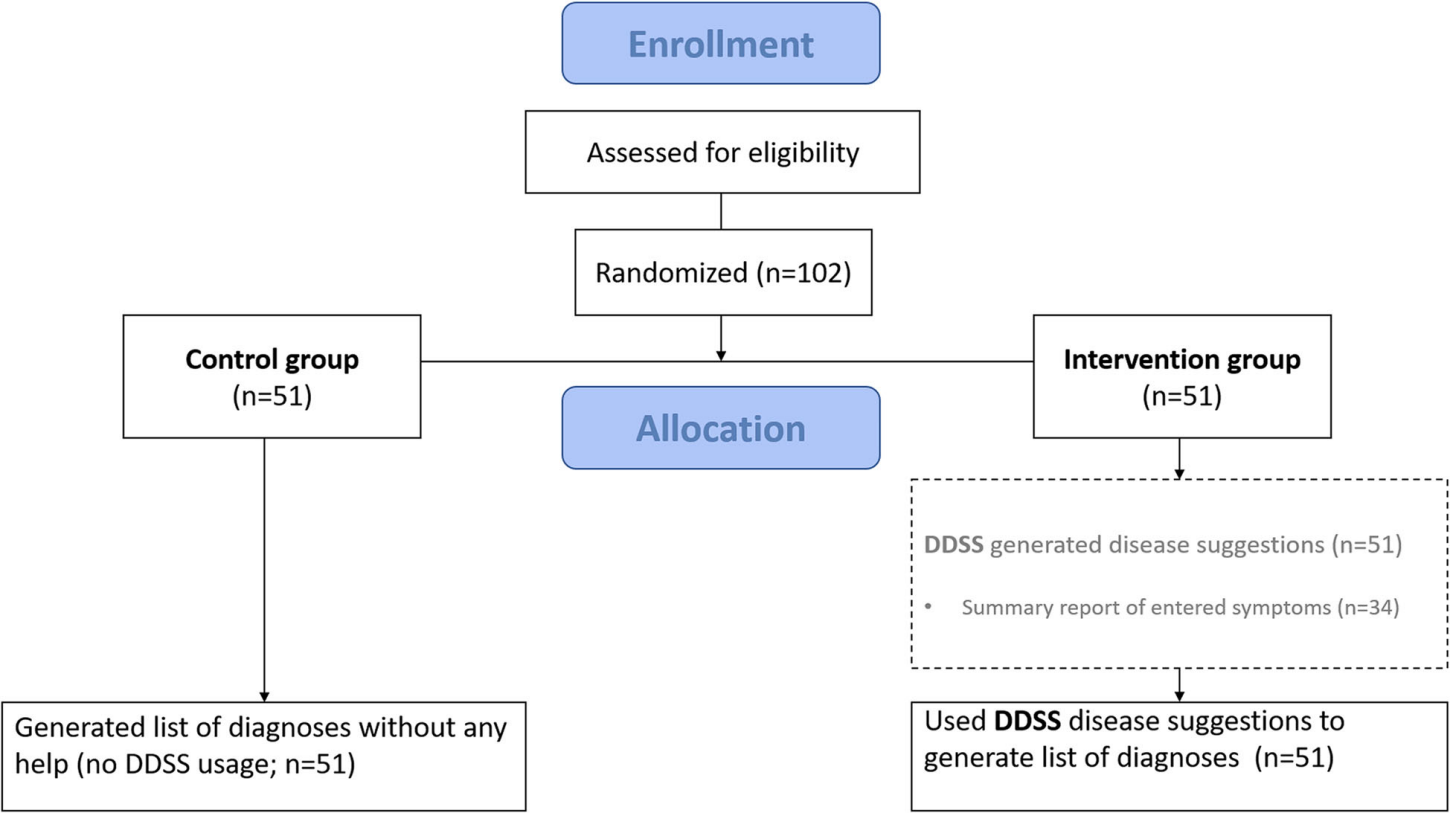
CONSORT flow diagram

We performed a manufacturer-independent unblinded parallel-group randomized controlled trial, which was approved by the ethics committee of the medical faculty of the University of Erlangen-Nürnberg, Germany (423_18 B, date of approval: 22 November 2018). Refer to Figure S1 for this study’s Consolidated Standards of Reporting Trials-eHealth checklist [23]. Participants were provided with 3 case vignettes (1st case: granulomatosis with polyangiitis [24], 2nd case: rheumatoid arthritis [25], 3rd case: systemic lupus erythematosus [26] from the public online learning center and Rheum2Learn section of the American College of Rheumatology. By selecting freely available cases, we wanted to ensure possible future comparisons of other DDSS systems. The information quality of the selected cases has been ensured by the American College of Rheumatology. We hoped to cover a case-mix including three representative rheumatic diseases for which there’s a varying degree of awareness and a varying requirement of clinical skills and knowledge integration capabilities among medical students. The exact choice of the three cases was informed by prior informal discussions between medical students and the first author (JK). Students stated that rheumatoid arthritis and secondly systemic lupus erythematosus were typical rheumatic diseases, whereas they did not mention granulomatosis with polyangiitis. Case vignettes were translated into German, and information concerning laboratory results or physical examination findings were deleted and not presented to students, so that only information on case history was available to students. Participants were randomized 1:1 to an intervention group (IG) (using DDSS) or control group (CG) (no-help) by computer-generated block randomization with a block size of 2 (Fig. 1). All participants were instructed to list up to five likely diagnoses and record the case completion time. IG participants had access to the DDSS, which provides up to 5 disease suggestions, while creating the ordered diagnostic hypotheses list. Afterwards, participants were asked to complete an evaluation form including (1) demographic data, (2) medical studies grade point average (1 = best grade, 6 = worst grade), (3) self-perceived knowledge about rheumatic diseases (5-point Likert scale), and (4) willingness to use the DDSS in clinical routine (yes/no). Intervention group participants were additionally asked to assess the helpfulness of Ada to create a list of differential diagnoses (5-point Likert scale) and the app usability (5-point Likert scale).

### Study participants

The inclusion criteria for participants were (1) students having completed at least one semester of internal medicine and (2) age ≥ 18 years old (no upper age limit given). The exclusion criterion was (1) participants unwilling or unable to comply with the protocol. All participants provided written informed consent before participating. Participants were not paid for their study participation. Participants were openly recruited from a university hospital between November 7, 2019, and May 25, 2020.

### Software and hardware

Study accounts were created on 6th generation Ipad devices (iOS 13.4.1, Apple Inc.) for the study, and IG participants received a brief explanation of how to use the DDSS. Namely, the artificial intelligence–driven Ada chatbot app asks a varying number of questions depending on the previous answers given and provides up to 5 precise disease suggestions, their probability, triage advice, and additional information. The DDSS software was regularly updated along the course of the study to ensure functionality, and as a result, multiple versions were used in the intervention group (versions 2.49.2, 2.49.3, 2.49.4, 3.0.0, 3.0.2, 3.0.3, 3.1.0, 3.1.2, 3.2.0, 3.3.0, 3.4.0, 3.5.0, Ada Health). Study personnel was present to assist the participants if needed. The DDSS generates a pdf summary report containing the symptoms entered by the user, negated symptom questions, and the list of suggested diagnoses (Figure S2). Due to a software bug, this feature was available only intermittently along the study course, and summary reports could be generated and saved for 34/51 participants in the intervention group. DDSS diagnoses and probabilities were recorded directly after case completion for all 51 participants so that the primary outcome analysis for the app output was not dependent on the availability of the pdf reports.

### Statistical analysis

We summarized the participant characteristics and outcomes using appropriate descriptive statistics as per data type. In the descriptive and exploratory analyses of the correct diagnosis rank, a rank of 6 was assigned to the instances in which no correct diagnosis was provided. Diagnostic accuracy was described as the overall proportion of correct diagnoses for each case and as the cumulative proportion of correct diagnoses across the ranks of suggested diagnoses where the fifth rank indicates the overall proportion of correct diagnoses. For the cumulative proportions, we also estimated 95% confidence intervals based on binomial distribution using the Wilson score method.

Since each participant evaluated three cases, we used mixed-effects regression for between-group comparisons with the case indicator included as a random-effects term in all models. The overall effect of the intervention was assessed using a mixed-effects logistic regression where the binary status of overall correct diagnosis was the dependent variable and the study group was the fixed effect. Exponentiated coefficient of the group term from this model indicated the overall odds ratio of obtaining a correct diagnosis across all cases. Time to solve cases was compared between the groups using a linear mixed-effects model using the same terms but time as the dependent variable. We explored the association between participant characteristics and correct diagnosis regardless of the study group using separate mixed-effects models including the semester, grade point average, and self-rated rheumatology knowledge as continuous fixed effects.

Along with a suggested diagnosis, the DDSS also outputs an estimated probability of the diagnosis given the symptoms entered. We calculated the mean of these estimated probabilities and 95% bootstrap confidence intervals for correct and incorrect diagnoses separately to explore whether at each diagnosis rank, a higher average probability was estimated by the DDSS for correctly suggested diagnoses.

We calculated Spearman rank correlations between the percentage of symptoms described in the case vignettes that were correctly extracted by the student and the rank of correct diagnoses obtained from the DDSS and participants in the intervention group. In this analysis, a rank of 6 was assigned when all the suggested diagnoses were incorrect. We compared the willingness to use the DDSS in future practice in the intervention vs. control groups using a chi-squared test.

All analyses were undertaken using the open-source R software v 4.0.1 (R Foundation for Statistical Computing, Vienna, Austria) running under RStudio IDE v 1.2.1 with the “tidyverse,” “binom,” and “lmerTest” packages. Two-tailed *p* values less than 0.05 were considered significant.

## Results

Between November 7, 2019, and May 25, 2020, 102 students participated in the study. The baseline characteristics were well balanced across the study groups (Table 1).

**Table 1:** Baseline characteristics overall and by group

		Intervention	Control	Overall
*N*		51	51	102
Male	*N* (%)	22 (43.1)	21 (41.2)	43 (42.2)
Female	*N* (%)	29 (56.9)	30 (58.8)	59 (57.8)
Age, years	Mean (SD)	25.3 (3.6)	24.6 (2.6)	25.0 (3.1)
Semester	Median (IQR)	9.0 (7.0–9.5)	9.0 (7.0–10.0)	9.0 (7.0–10.0)
Grade point average	Median (IQR)	2.0 (1.8–2.1)	2.0 (1.8–2.2)	2.0 (1.8–2.2)
Rheumatology knowledge	Median (IQR)	2.0 (1.0–3.0)	2.0 (2.0–3.0)	2.0 (1.0–3.0)

### Student and DDSS diagnoses

A total of 1362 differential diagnoses were recorded. The total number of provided diagnoses by the CG, IG, and DDSS was 321, 416, and 625, respectively. The total number of provided unique diagnoses by the CG, IG, and DDSS was 56, 62, and 59, respectively. Figure S3 shows all the top diagnoses for the three cases by the two study groups and the DDSS.

### Correct diagnoses suggested by study groups, DDSS, and the effect of intervention

**Fig. 2:**
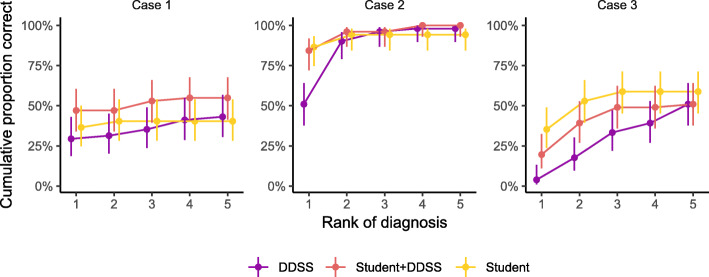
Cumulative proportion of correct diagnoses by rank

Table 2 and Fig. 2 show the cumulative proportion of correct diagnoses with 95% confidence intervals. The cumulative proportion describes the proportion of correct diagnoses up to a given rank of suggested diagnoses. The proportion of correct diagnoses for the first rank suggestion (and overall, i.e., 5th rank) in CG, IG, and DDSS was 37% (40%), 47% (55%), and 29% (43%) for the first case; 87% (94%), 84% (100%), and 51% (98%) in the second case; and 35% (59%), 20% (51%), and 4% (51%) in the third case, respectively. The overall median rank of correct diagnosis (IQR) was 2 (1–6) in the control group, 3 (1.5–4.5) in the intervention group, and 3 (2–5) for DDSS. In correspondence with this, the cumulative proportion of correct diagnoses plateaued in the 2nd and 3rd ranks in the control group in contrast with the 4th and 5th ranks in the intervention group and the DDSS (Fig. 2). The overall odds ratio for a correct diagnosis in the intervention group compared to the control group was 1.27 (95%CI 0.75 to 2.16, *p* = 0.380).

**Table 2:** Cumulative number and proportion of correct diagnoses by case and study group

Case	Rank	Control group (***n*** = 51)		Intervention group (***n*** = 51)		DDSS (***n*** = 51)	
		Correct	Proportion (95%CI)	Correct	Proportion (95%CI)	Correct	Proportion (95%CI)
1	1	19	0.37 (0.25 to 0.50)	24	0.47 (0.34 to 0.60)	15	0.29 (0.19 to 0.43)
	2	21	0.40 (0.28 to 0.54)	24	0.47 (0.34 to 0.60)	16	0.31 (0.20 to 0.45)
	3	21	0.40 (0.28 to 0.54)	27	0.53 (0.40 to 0.66)	18	0.35 (0.24 to 0.49)
	4	21	0.40 (0.28 to 0.54)	28	0.55 (0.41 to 0.68)	21	0.41 (0.29 to 0.55)
	5	21	0.40 (0.28 to 0.54)	28	0.55 (0.41 to 0.68)	22	0.43 (0.31 to 0.57)
2	1	45	0.87 (0.75 to 0.93)	43	0.84 (0.72 to 0.92)	26	0.51 (0.38 to 0.64)
	2	49	0.94 (0.84 to 0.98)	49	0.96 (0.87 to 0.99)	46	0.90 (0.79 to 0.96)
	3	49	0.94 (0.84 to 0.98)	49	0.96 (0.87 to 0.99)	49	0.96 (0.87 to 0.99)
	4	49	0.94 (0.84 to 0.98)	51	1.00 (0.93 to 1.00)	50	0.98 (0.90 to 1.00)
	5	49	0.94 (0.84 to 0.98)	51	1.00 (0.93 to 1.00)	50	0.98 (0.90 to 1.00)
3	1	18	0.35 (0.24 to 0.49)	10	0.20 (0.11 to 0.32)	2	0.04 (0.01 to 0.13)
	2	27	0.53 (0.40 to 0.66)	20	0.39 (0.27 to 0.53)	9	0.18 (0.10 to 0.30)
	3	30	0.59 (0.45 to 0.71)	25	0.49 (0.36 to 0.62)	17	0.33 (0.22 to 0.47)
	4	30	0.59 (0.45 to 0.71)	25	0.49 (0.36 to 0.62)	20	0.39 (0.27 to 0.53)
	5	30	0.59 (0.45 to 0.71)	26	0.51 (0.38 to 0.64)	26	0.51 (0.38 to 0.64)

**Fig. 3:**
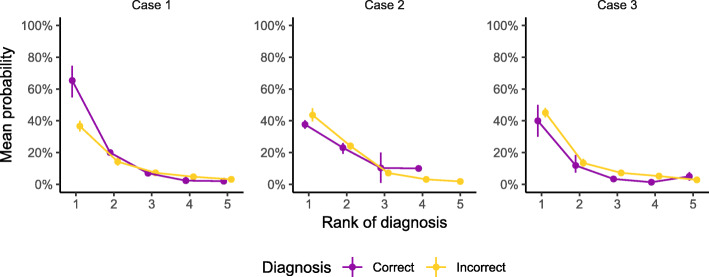
Mean DDSS-based diagnostic probabilities by case, diagnosis rank, and ground truth

Figure 3 depicts the mean and bootstrapped 95% confidence intervals of the diagnostic probabilities reported by the DDSS obtained by each user separately by the rank of suggested diagnosis, case, and whether the given diagnosis is correct or not. The plot shows that the mean probability given by the DDSS for a correct diagnosis was meaningfully higher than those of incorrect diagnoses only for the rank 1 diagnosis in case 1 whereas in a substantial number of situations, the mean probabilities reported by the DDSS for correct diagnoses were actually lower than those for incorrect diagnoses.

### Association of student characteristics and correct diagnosis

Students were more likely to find the overall correct diagnosis as seniority (OR per semester, 1.33, 95%CI 1.11 to 1.60) and self-rated rheumatology knowledge (OR per 1 point increase in rating 1.42, 95%CI 1.08 to 1.87) increased, whereas we did not find a conclusive association between the probability of making the correct diagnosis and grade point average (Table 3).

**Table 3:** Univariable associations between student characteristics and correct diagnosis

	OR (95%CI)	***p*** value
Semester	1.33 (1.11 to 1.60)	0.003
Grade point average	0.53 (0.26 to 1.07)	0.075
Self-rated rheumatology knowledge	1.42 (1.08 to 1.87)	0.011

### Symptoms entered into DDSS and correlation with correct diagnosis

All symptoms that were entered first in the DDSS group and their association with the correct diagnosis are displayed in Figure S4. Table 4 displays the mentioned key symptoms for the three case vignettes and the percentage of DDSS entry. Symptom entry order and completeness largely varied. In the first case, no participant entered, for example, “sinus infection,” “ear infection,” or “hemoptysis,” whereas all students stated “morning stiffness” while completing the second case. Figure S5 lists all symptoms entered into the DDSS and their entry order and the resulting top diagnosis for case 2. The symptoms were extracted from the 34 available summary reports. We found no significant correlation between the percentage of symptoms correctly extracted from the case vignettes and the rank of correct diagnosis (Table 5).

**Table 4:** Symptoms described in case vignettes and DDSS entry frequency (*n* = 34)

Symptoms, ***n***	Case 1, ***n*** (%)	Case 2, ***n*** (%)	Case 3, ***n*** (%)
1	Fever 33 (97.1)	Morning stiffness 34 (100.0)	Fatigue 31 (91.2)
2	Cough 33 (97.1)	Painful finger joints 25 (73.5)	Arthralgia 34 (100.0)
3	Arthralgia 32 (94.1)	Painful wrist joints 11 (32.4)	Morning stiffness 23 (67.6)
4	Nasal congestion 16 (47.1)	Fatigue 30 (88.2)	Joint swelling 16 (47.1)
5	Hemoptyses 0 (0.0)	Reduced flexibility in the wrist 5 (14.7)	Neck rash 24 (70.1)
6	Sinusitis 0 (0.0)	Reduced finger flexibility 20 (58.8)	Face rash 11 (32.4)
7	Ear infections 0 (0.0)		
8	Fatigue 23 (67.7)		
9	Dyspnea on exertion 31 (91.2)		
10	Painful eye 17 (50.0)		
11	Red eye 25 (73.5)

**Table 5:** Correlation between the percentage of symptoms extracted and the rank of correct diagnoses from DDSS and the rank of correct diagnoses in the intervention group

Case	DDSS		Student + DDSS	
	Rho	***p*** value	Rho	***p*** value
1	− 0.01	0.92	− 0.03	0.82
2	− 0.05	0.72	0.01	0.97
3	− 0.12	0.40	− 0.06	0.68

### Time to solve cases and relationship between correct diagnosis

**Fig. 4:**
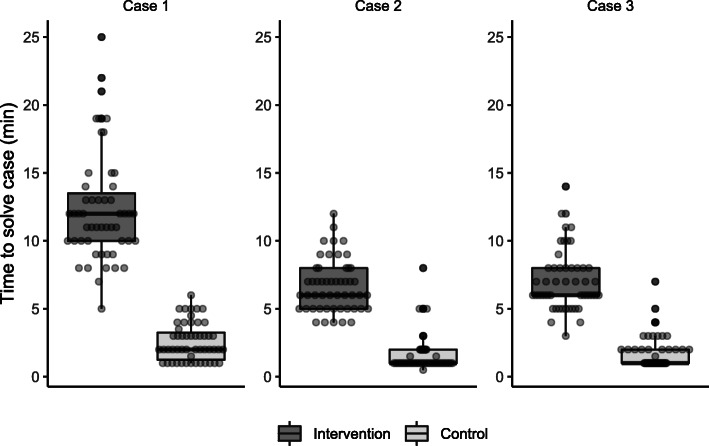
Time to solve cases by case and study group

Figure 4 summarizes the time elapsed to solve each case in each study group by correct diagnosis. Although the time to solve cases was shorter for correct diagnoses, this was heterogenous across cases, and overall, there was essentially no difference in the time to solve cases for correct or incorrect diagnoses. The odds ratio for a correct diagnosis by time was 1.00 (95%CI 0.95 to 1.06, *p* = 0.940). The time to solve the cases was substantially longer in the IG compared to CG. On average, the IG required an approximate 7 min to solve the cases and to operate the DDSS (6.65, 95%CI 6.09 to 7.22, *p* < 0.001). On average, case 1 took the longest to complete (CG 3 min; IG 12 min) followed by cases 2 and 3 (CG 2 min; IG 7 min).

### DDSS adherence and symptom entry order

The IG group stated the same top diagnosis as suggested by the DDSS 58.8% (30/51), 60.8% (31/51), and 51.0% (26/51) of the time for the three cases, respectively. The correct DDSS D1 (top diagnosis) was accepted in 100.0% (15/15), 96.2% (25/26), and 50.0% (1/2) for the three cases, respectively. On the other hand, the IG group also accepted false suggestions. Looking at Fig. S3 and the first case, the DDSS top diagnosis suggestion “pneumonia” was accepted as the top diagnosis by 55.6% (5/9) students in the IG, whereas no one stated it in the CG. For the second case, the DDSS top diagnosis suggestion “Felty syndrome” was accepted as the top diagnosis by 31.6% (6/19) and no one stated “Felty syndrome” as a top diagnosis in the CG. For the third case, the DDSS top diagnosis suggestion “fibromyalgia” was accepted as the top diagnosis by 37.5% (6/16) and again no one stated “fibromyalgia” as a top diagnosis in the CG. Furthermore, Figures S4 and S5. suggest that symptom entry order, entry completeness, and respective diagnostic suggestions varied greatly between participants.

### Student attitudes toward usefulness and use of the DDSS for future practice

In CG (without having used the DDSS), 46/51 (90.2%) students stated that they could consider using the DDSS in their future clinical work compared to 31/51 (60.8%) students in the IG (after having used the DDSS), indicating a significantly reduced proportion of willingness to use the DDSS in the IG (*p* = 0.001). Usefulness was rated with a mean of 3.2 (SD 1.0) and ease of use with an average of 2.0 (SD 1.2) (5 = very useful/very hard) in the IG.

## Discussion

To the best of our knowledge, this is the first randomized controlled trial aimed at investigating the contribution of an AI-based DDSS [18] to diagnostic accuracy to solve rheumatic case vignettes by future physicians. Thirty years ago, Moens et al. concluded in their review that rheumatology is a suitable domain for computer-assisted diagnosis and presented various promising systems [22]. Furthermore, the authors concluded that most of the evaluation studies were carried out with some involvement of the DDSS developers. Importantly, this study has been carried out without any involvement of the DDSS developers, in contrast to currently published DDSS evaluations [18, 21]. Furthermore, the usage of publicly available case vignettes allows future DDSS comparisons.

The main finding of this study is that DDSS usage did not make a significant contribution to the already developed diagnostic skills of medical students. The overall diagnostic accuracy was very case-dependent. One might be seriously concerned about additional confusion introduced by a DDSS in the hands of an inexperienced diagnostician for a number of reasons. Firstly, the overall number of diagnoses suggested by DDSS was almost twice the number of diagnoses suggested by the unaided students in the control group (625 vs. 321), indicating that a DDSS tends to suggest as many possible diagnoses as requested by the user regardless of their true relevance. Secondly, the median rank of correct diagnoses in the control group was lower (2 vs. 3 in the intervention group). Finally, the probabilities suggested by the DDSS were lower for correct diagnoses than those for incorrect diagnoses in most of the cases (Fig. 3), potentially misleading users.

In 2014, Alder et al. identified 25 computer-based diagnostic systems for rheumatology showing moderate to excellent performance and concluded that the external validation process was in general underappreciated, and none of the systems seemed to have succeeded in daily practice [27]. Interestingly, a similar trial published in 1989 by McCrea et al., including 119 medical students diagnosing 10 rheumatology cases, showed more positive results [28]. Another DDSS specifically developed for RMDs using fuzzy cognitive map technique showed a diagnostic accuracy of 87% in a validation study with 15 cases [29]. A diagnostic support system for specialists in internal medicine suggested the correct diagnosis in 48 of 50 cases (96%) using case vignettes [30].

Interestingly, the DDSS group included most differential diagnoses, and we found many students to click on suggested diagnoses to view associated symptoms. This was however not properly measured. Despite the medical background and clear instructions to enter the symptoms as precisely as possible, key symptoms were not at all entered or were entered incorrectly. For example, the typical butterfly rash described in the third case vignette was entered only by 32% (11/34) patients. It seems unlikely that older, multimorbid patients without a medical background will enter their symptoms more precisely and end up with a higher diagnostic accuracy. A first rheumatology-specific study with 34 patients using symptom checkers showed that only 4 out of 21 patients with inflammatory arthritis were given the first diagnosis of rheumatoid arthritis or psoriatic arthritis [31]. Importantly, we could recently show that the same DDSS, when used by patients with inflammatory rheumatic diseases, displayed the correct diagnoses among the first and overall disease suggestions in 17% (9/54) and 26% (14/54) of the time [32]. Surprisingly, we found no correlation between the proportion of symptoms correctly extracted and the DDSS diagnostic accuracy. As expected, seniority in medical education and self-rated rheumatology knowledge were associated with the likelihood of suggesting an overall correct diagnosis.

Completing the cases using the DDSS took significantly longer. This is reflected by the approximate 30% decrease of students willing to use the DDSS in their future clinical work life after having used the DDSS. Previous research showed that using DDSS during a consultation is feasible without increasing consultation time [14]. Perceived usefulness was average, and students generally perceived the DDSS as easy to use. The integration of innovative digital tools such as DDSS into routine medical education could likely increase student interest in the discipline of rheumatology, similarly to online ultrasound modules [33].

Limitations of the study include the unblinded pilot study design and relatively [18, 20, 29] small sample size. The trial was registered retrospectively, and we did not pre-specify an effect size nor carried out a sample size calculation. However, such “AI”-based applications commonly claim an individual level accuracy, which we believe should be apparent in a modestly sized trial such as ours, where the reduced willingness to use the app in the future was clearly apparent in the intervention group. The results, on the other hand, reflect the accuracy in the diagnosis of three diseases which were based on case vignettes and not real patients. Although this standardizes the case definitions, it limits the generalizability to the overall rheumatic diseases and also overlooks within-disease variations. Furthermore, the participants were medical students; therefore, the results cannot also be generalized to physicians. We did not record which medical students already received training in rheumatology. Due a technical flaw of the DDSS, summary reports could not be generated for all participants. The influence of DDSS software updates on diagnostic accuracy was not analyzed in this study; however, since the intervention and control groups were recruited in parallel over time, a radical improvement in diagnostic accuracy across newer versions would have likely produced a signal in favor of the DDSS in the results.

To identify more directly the influence of DDSS on the diagnostic approach, future study subjects should record their initial differential diagnoses and their diagnoses after considering the DDSS suggestions. Furthermore, it seems worthwhile to let subjects rate their perceived diagnostic accuracy. Specific DDSS training for physicians and patients might improve diagnostic results and user acceptance. Furthermore, strategies need to be identified to reduce burdens for physicians to integrate DDSS into their clinical routine. SCs could be used by patients prior to an appointment and the SC data should automatically be made available to the physician to prevent redundant work and prompt text modules for medical reports.

## Conclusion

DDSS usage did not significantly improve the students’ own diagnostic skills, which were superior to the DDSS accuracy. The misleading DDSS diagnostic probabilities and necessary DDSS entering time represent further major caveats for their implementation into clinical routine. Although generally easy to use, the significantly lower number of students that were willing to use the DDSS in their future clinical work in the intervention group (having used the DDSS) suggests an unfavorable outlook.

## Supplementary Information

**Additional file 1: Figure S1.** Consolidated Standards of Reporting Trials-eHealth checklist.

**Additional file 2: Figure S2.** Exemplary DDSS summary report for a second case.

**Additional file 3: Figure S3.** Top diagnoses for all cases and study groups.

**Additional file 4: Figure S4.** All symptoms entered first in the DDSS group and their association with a correct diagnosis.

**Additional file 5: Figure S5.** All symptoms entered and their entry order into the DDSS for case 2.

## Data Availability

Data are available from the corresponding author on reasonable request.

## Abbreviations

AI: Artificial intelligence
CG: Control group
DDSS: Diagnostic decision support system
GP: General practitioners
IG: Intervention group
RMDs: Rheumatic musculoskeletal diseases
SCs: Symptom checkers
