# Supplementary material for: Accuracy and usability of a diagnostic decision support system in the diagnosis of three representative rheumatic diseases: a randomized controlled trial among medical students
Source: Arthritis Res Ther. 2021 Sep 6;23:233. doi: 10.1186/s13075-021-02616-6 (PMC8420018; doi:10.1186/s13075-021-02616-6)
Supplement: Supplementary file 2 — Additional file 2: Figure S2. Exemplary DDSS summary report for a second case. [file 13075_2021_2616_MOESM2_ESM.pdf]

Assessment Report

morning stiffness

Case 2, Female, 1999

Reported symptoms

Symptoms reported as present

- Morning stiffness
  - time since onset: one month to one year
- Painful wrist
  - time since onset: one month to one year
  - activity: relieves
  - laterality: bilateral
  - intensity: moderate
- Fatigue
  - time since onset: one month to one year
- Pain in more than one joint
  - time since onset: one month to one year
  - pain on exertion: relieves
- Joint pain in fingers
  - time since onset: one month to one year
  - activity: relieves
  - laterality: bilateral
  - intensity: moderate
- Tenderness at finger joints
  - laterality: bilateral
- Reduced mobility of the fingers

Symptoms reported as absent

- Generalised muscle pain
- Prolonged nighttime sleep
- Difficulty swallowing
- Difficulty breathing
- Lump under the skin over a joint
- Change in nail growth
- Knee pain
- Cold skin on arms or legs
- Spots on the arm
- Foot pain
- Neck pain
- Hardened skin on the arm
- All-over darkening of skin
- Tense muscles all over
- Urinating less
- Spots on the leg
- Bunion
- Smoker
- Pregnant
- Diabetes
- High blood pressure

Symptoms reported as unsure of

- Swollen finger joint

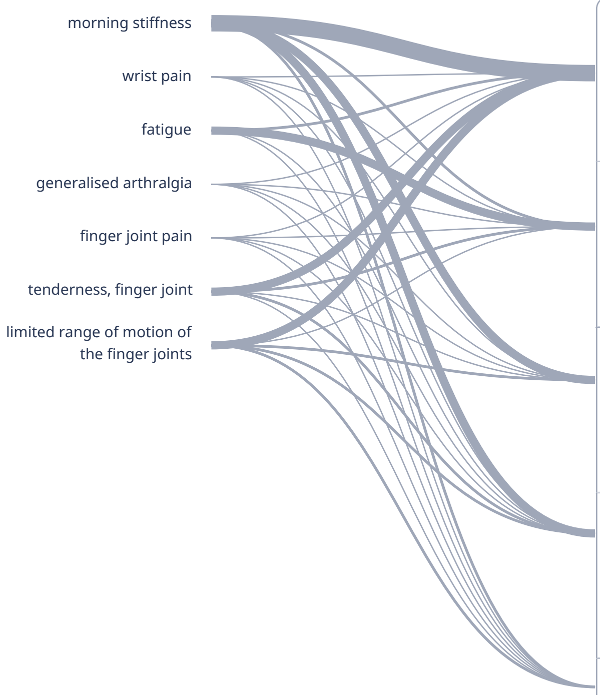

Rheumatoid arthritis

Seek medical advice

4 out of 10 people with these symptoms had this condition.

Diffuse cutaneous systemic sclerosis

Seek medical advice

1 out of 10 people with these symptoms had this condition.

Osteoarthritis, generalised

Seek medical advice

8 out of 100 people with these symptoms had this condition.

Felty syndrome

Seek medical advice

8 out of 100 people with these symptoms had this condition.

Psoriatic arthritis

Seek medical advice

4 out of 100 people with these symptoms had this condition.

Next Steps

People with symptoms similar to yours do not usually require urgent medical care. You should seek advice from a doctor though, within the next 2-3 days. If your symptoms get worse, or if you notice new symptoms, you may need to consult a doctor sooner.

1 Rheumatoid arthritis Seek medical advice

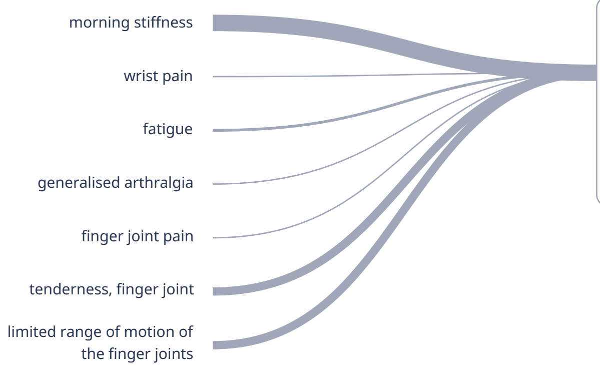

Rheumatoid arthritis

Seek medical advice

4 out of 10 people with these symptoms had this condition.

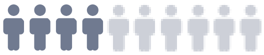

Description

Rheumatoid arthritis is an inflammatory disease of the joints. This is caused by an autoimmune reaction, which means that the immune system mistakenly forms antibodies (proteins which fight infections) and begins to attack the body tissues. Women are affected more commonly than men, and most people are diagnosed between 35 and 50 years age. The symptoms of rheumatoid arthritis are pain and swelling in the joints, especially the small joints in the hands and feet. Rheumatoid arthritis is diagnosed by the symptoms, physical examination and a blood test. Rheumatoid arthritis is managed with pain medication and medications to slow down the immune system, but there is not specific cure for this condition. Rheumatoid arthritis usually gets slowly worse over time, and can cause significant joint damage. Early treatment can help prevent some joint damage.

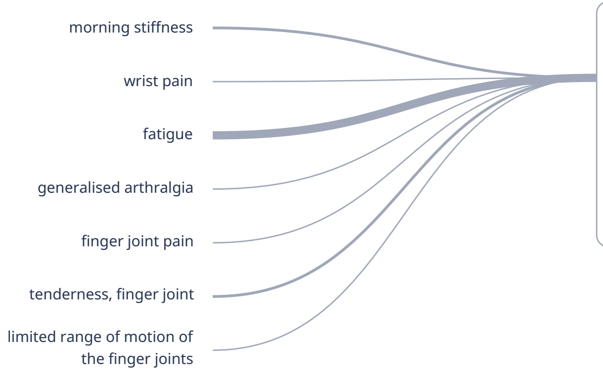

Diffuse cutaneous systemic sclerosis

Seek medical advice

1 out of 10 people with these symptoms had this condition.

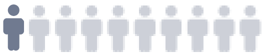

Description

Diffuse scleroderma, or diffuse cutaneous systemic sclerosis, is an autoimmune condition where the immune system mistakenly attacks connective tissue, which supports and connects other body organs and tissues. The precise causes of diffuse scleroderma are not clear, but it is more commonly seen in women. Typical symptoms can include hardening of the skin, joint pain, breathing and digestive problems. Physical examination, blood tests, and a skin biopsy are usually performed to establish the diagnosis. Although there is no cure for diffuse scleroderma, treatment aims to relieve symptoms and prevent complications. The outlook of the condition varies widely depending on how the affected person responds to treatments.

3 Osteoarthritis, generalised Seek medical advice

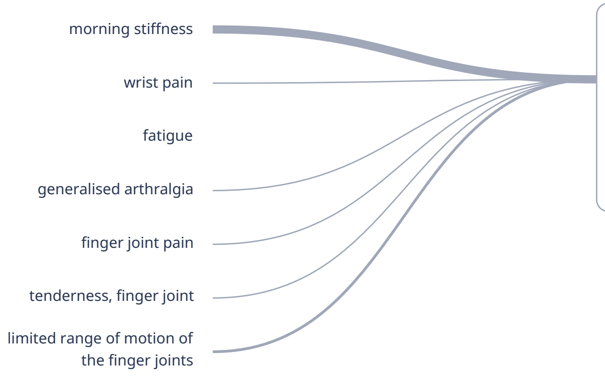

Osteoarthritis, generalised

Seek medical advice

8 out of 100 people with these symptoms had this condition.

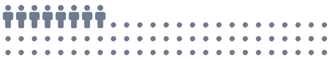

Description

Generalised osteoarthritis is damage to bones and cartilage in multiple joints of the body. This common condition occurs in people as they age, often affecting knees, hips, fingers, feet and back. It is mostly due to wear and tear on the joints over time, although genes, injuries and increased body weight also contribute to the development of osteoarthritis. The typical symptom is a deep, achy pain felt in the affected joints. Osteoarthritis ranges in severity from requiring no treatment, to requiring pain medication or joint replacement. Although osteoarthritis gradually becomes worse over time, most people learn to manage their symptoms.

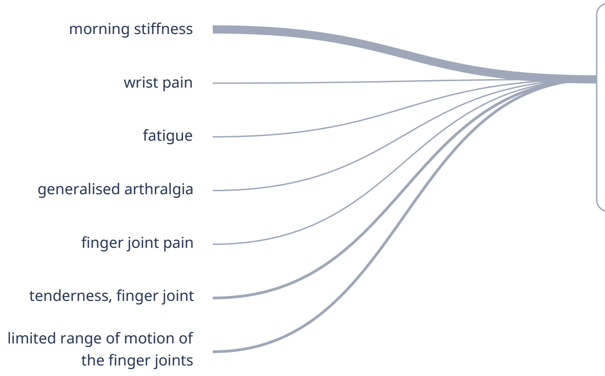

Felty syndrome

Seek medical advice

8 out of 100 people with these symptoms had this condition.

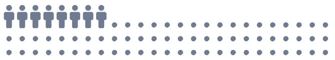

Description

Felty's syndrome is a rare condition characterized by three components: rheumatoid arthritis (a chronic inflammatory joint condition), an enlarged spleen, and a low white blood cell count. Symptoms include joint swelling and pain, frequent infections, and feeling generally unwell. The diagnosis is based on a physical exam, blood tests, and an ultrasound to assess the size of the spleen. Since there is no cure, treatment focuses on relieving symptoms and preventing more infections. In some cases, the spleen has to be removed. Because affected people have a higher risk for recurrent infections, close medical monitoring is usually necessary.

morning stiffness

wrist pain

fatigue

generalised arthralgia

finger joint pain

tenderness, finger joint

limited range of motion of the finger joints

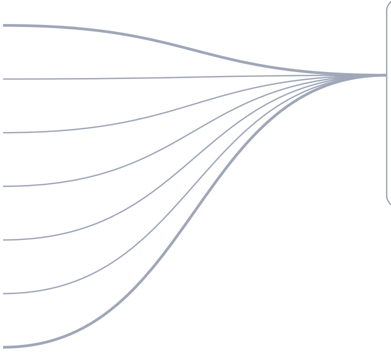

Psoriatic arthritis

Seek medical advice

4 out of 100 people with these symptoms had this condition.

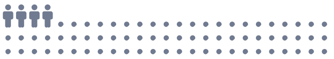

Description

Psoriatic arthritis is an inflammation of the joints. It is caused by an overactive immune system. The cause is similar to the skin condition psoriasis. Most people experience skin psoriasis before they get joint pain. Some people may develop skin symptoms later on or no skin symptoms at all. This condition is genetic. It usually affects young adults but can affect people of all ages. Symptoms may include swollen and painful joints in the hands and feet. The diagnosis is made based on the symptoms, blood tests, and X-rays or scans of the joints. Early and adequate treatment is important to prevent joint damage. Treatment includes medicines to control the immune system and prevent further joint damage. It is a life-long condition, but most people manage to control the symptoms.
