# Supplementary material for: Accuracy and usability of a diagnostic decision support system in the diagnosis of three representative rheumatic diseases: a randomized controlled trial among medical students
Source: Arthritis Res Ther. 2021 Sep 6;23:233. doi: 10.1186/s13075-021-02616-6 (PMC8420018; doi:10.1186/s13075-021-02616-6)
Supplement: Supplementary file 3 — Additional file 3: Figure S3. Top diagnoses for all cases and study groups. [file 13075_2021_2616_MOESM3_ESM.pdf]

# 1st Case: Granulomatosis with Polyangiitis

| Top Diagnosis | **Control group** (N=51) | **Intervention group** (N=51) | **DDSS** (N=51) |
|---|---|---|---|
| | n (%) | n (%) | n (%) |
| Acute bronchitis | 0 (0.0) | 3 (5.9) | 9 (17.7) |
| Acute lymphoblastic leukemia | 0 (0.0) | 1 (2.0) | 0 (0.0) |
| Acute myeloid leukemia | 0 (0.0) | 2 (3.9) | 1 (2.0) |
| Acute Sarcoidosis | 1 (2.0) | 1 (2.0) | 1 (2.0) |
| Alpha-1 antitrypsin deficiency | 0 (0.0) | 1 (2.0) | 0 (0.0) |
| Bacterial conjunctivitis | 0 (0.0) | 1 (2.0) | 0 (0.0) |
| Bronchiectasis | 0 (0.0) | 0 (0.0) | 1 (2.0) |
| Chronic obstructive pulmonary disease | 0 (0.0) | 0 (0.0) | 3 (5.9) |
| Chronic sinusitis | 1 (2.0) | 0 (0.0) | 0 (0.0) |
| Churg-Strauss syndrome | 0 (0.0) | 4 (7.8) | 5 (9.8) |
| CMV infection | 1 (2.0) | 0 (0.0) | 0 (0.0) |
| Conjunctivitis | 0 (0.0) | 1 (2.0) | 1 (2.0) |
| Dermatomyositis | 1 (2.0) | 0 (0.0) | 0 (0.0) |
| Flu | 0 (0.0) | 3 (5.9) | 1 (2.0) |
| Granulomatosis with Polyangiitis | 19 (37.3) | 24 (47.1) | 15 (29.4) |
| Hordeolum | 0 (0.0) | 0 (0.0) | 1 (2.0) |
| Infectious arthritis | 1 (2.0) | 0 (0.0) | 0 (0.0) |
| Kawasaki syndrome | 1 (2.0) | 0 (0.0) | 0 (0.0) |
| Lung cancer | 1 (2.0) | 0 (0.0) | 0 (0.0) |
| Pleural empyema | 0 (0.0) | 0 (0.0) | 1 (2.0) |
| Pneumonia | 0 (0.0) | 5 (9.8) | 9 (17.7) |
| Post streptococcal infection | 1 (2.0) | 0 (0.0) | 0 (0.0) |
| Reactive arthritis | 9 (17.7) | 0 (0.0) | 0 (0.0) |
| Reiter's disease | 1 (2.0) | 0 (0.0) | 0 (0.0) |
| Respiratory infection | 1 (2.0) | 1 (2.0) | 0 (0.0) |
| Rheumatic fever | 5 (9.8) | 0 (0.0) | 0 (0.0) |
| Rheumatoid arthritis | 2 (3.9) | 1 (2.0) | 1 (2.0) |
| Septic granulomatosis | 0 (0.0) | 1 (2.0) | 1 (2.0) |
| Small vessel vasculitis | 1 (2.0) | 0 (0.0) | 0 (0.0) |
| Spondyloarthritis | 1 (2.0) | 0 (0.0) | 0 (0.0) |
| Systemic lupus erythematosus | 1 (2.0) | 1 (2.0) | 0 (0.0) |
| Vasculitis | 1 (2.0) | 0 (0.0) | 0 (0.0) |
| Viral respiratory infection | 2 (3.9) | 1 (2.0) | 0 (0.0) |
| Viral sinusitis | 0 (0.0) | 0 (0.0) | 1 (2.0) |

Green: Diagnoses accepted as corrected; Pink: Most frequent top diagnosis; Turquoise: 2nd most frequent top diagnosis; Grey: 3rd most frequent top diagnosis.

## 2nd Case: Rheumatoid Arthritis

| Top Diagnosis | Control group (N=51) n (%) | Intervention group (N=51) n (%) | DDSS (N=51) n (%) |
|---|---|---|---|
| Arthralgia | 0 (0.0) | 1 (2.0) | 0 (0.0) |
| Felty syndrome | 0 (0.0) | 6 (11.8) | 19 (37.3) |
| Fibromyalgia | 0 (0.0) | 0 (0.0) | 4 (7.8) |
| Snapping finger | 0 (0.0) | 0 (0.0) | 1 (2.0) |
| Generalized osteoarthritis | 4 (7.8) | 1 (2.0) | 0 (0.0) |
| juvenile idiopathic arthritis | 1 (2.0) | 0 (0.0) | 0 (0.0) |
| Osteoarthritis | 0 (0.0) | 0 (0.0) | 1 (2.0) |
| Psoriatic arthritis | 1 (2.0) | 0 (0.0) | 0 (0.0) |
| Rheumatoid arthritis | 45 (88.2) | 43 (84.3) | 26 (51.0) |

Green: Diagnoses accepted as corrected; Pink: Most frequent top diagnosis; Turquoise: 2nd most frequent top diagnosis; Grey: 3rd most frequent top diagnosis.

## 3rd Case: Systemic Lupus Erythematosus

| Diagnosis | Control group (N=51)<br>n (%) | Intervention group (N=51)<br>n (%) | DDSS (N=51)<br>n (%) |
|---|---|---|---|
| Adult onset still disease | 1 (2.0) | 1 (2.0) | 0 (0.0) |
| Dermatomyositis | 7 (13.7) | 0 (0.0) | 0 (0.0) |
| Felty syndrome | 0 (0.0) | 0 (0.0) | 3 (5.9) |
| Fibromyalgia | 0 (0.0) | 6 (11.8) | 16 (31.4) |
| Gout | 1 (2.0) | 0 (0.0) | 0 (0.0) |
| Hodgkin lymphoma | 0 (0.0) | 1 (2.0) | 1 (2.0) |
| Lyme disease | 1 (2.0) | 0 (0.0) | 1 (2.0) |
| Melasma | 0 (0.0) | 0 (0.0) | 1 (2.0) |
| Mixed connective tissue disease | 0 (0.0) | 1 (2.0) | 3 (5.9) |
| Mixed cryoglobulinemia | 0 (0.0) | 1 (2.0) | 1 (2.0) |
| Psoriatic arthritis | 20 (39.2) | 18 (35.3) | 7 (13.7) |
| Rheumatoid arthritis | 3 (5.9) | 13 (25.5) | 16 (31.4) |
| Systemic lupus erythematosus | 18 (35.3) | 10 (19.6) | 2 (3.9) |

Green: Diagnoses accepted as corrected; Pink: Most frequent top diagnosis; Turquoise: 2nd most frequent top diagnosis; Grey: 3rd most frequent top diagnosis.
