# Supplementary material for: Accuracy and usability of a diagnostic decision support system in the diagnosis of three representative rheumatic diseases: a randomized controlled trial among medical students
Source: Arthritis Res Ther. 2021 Sep 6;23:233. doi: 10.1186/s13075-021-02616-6 (PMC8420018; doi:10.1186/s13075-021-02616-6)
Supplement: Supplementary file 4 — Additional file 4: Figure S4. All symptoms entered first in the DDSS group and their association with a correct diagnosis. [file 13075_2021_2616_MOESM4_ESM.pdf]

1st Case: Granulomatosis with Polyangiitis

**First symptom entered**	**n (%)**	**associated with correct diagnosis**; n (%)
Cough	2 (3.9)	0 (0.0)
Eye pain	1 (2.0)	0 (0.0)
Fever	21 (41.2)	3 (14.3)
General feeling of illness	2 (3.9)	1 (50.0)
Pain in more than one joint	14 (27.5)	8 (57.1)
Pain in the paranasal sinuses	2 (3.9)	1 (50.0)
Recurring infection of the respiratory tract	4 (7.8)	2 (50.0)
Red eye	4 (7.8)	0 (0.0)
Respiratory problems	1 (2.0)	0 (0.0)

Pink: First symptom associated most frequently with corrected diagnosis; Turquoise: First symptom associated 2nd most frequently with correct diagnosis; Grey: First symptom associated 3rd most frequently with correct diagnosis.

2nd Case: Rheumatoid Arthritis

**First symptom entered**	**n (%)**	**associated with correct diagnosis; n (%)**
Hand pain	9 (17.7)	4 (44.4)
Joint pain in fingers	8 (15.7)	1 (12.5)
Morning stiffness	2 (3.9)	2 (100.0)
Pain in more than one joint	9 (17.7)	7 (77.8)
Reduced flexibility in multiple joints	1 (2.0)	0 (0.0)
Reduced flexibility in one´s fingers	8 (15.7)	6 (75.0)
Reduced flexibility in the wrists	3 (5.9)	3 (100.0)
Wrist pain	11 (21.6)	3 (27.3)

Pink: First symptom associated most frequently with corrected diagnosis; Turquoise: First symptom associated 2nd most frequently with correct diagnosis; Grey: First symptom associated 3rd most frequently with correct diagnosis.

3rd Case: Systemic Lupus Erythematosus

**First symptom entered**	**n (%)**	**associated with correct diagnosis**; n (%)
Fatigue	9 (17.7)	0 (0.0)
Morning stiffness	1 (2.0)	0 (0.0)
Neck exanthema	5 (9.8)	1 (20.0)
Pain in more than one joint	36 (70.6)	1 (2.8)

Pink: First symptom associated most frequently with corrected diagnosis; Turquoise: First symptom associated 2nd most frequently with correct diagnosis; Grey: First symptom associated 3rd most frequently with correct diagnosis.
