# Supplementary material for: Accuracy and usability of a diagnostic decision support system in the diagnosis of three representative rheumatic diseases: a randomized controlled trial among medical students
Source: Arthritis Res Ther. 2021 Sep 6;23:233. doi: 10.1186/s13075-021-02616-6 (PMC8420018; doi:10.1186/s13075-021-02616-6)
Supplement: Supplementary file 5 — Additional file 5: Figure S5. All symptoms entered and their entry order into the DDSS for case 2. [file 13075_2021_2616_MOESM5_ESM.pdf]

Participant number	1	2	3	4	5	6	7	8	9	10	11	12	13	14	15	16	17	18	19	20	21	22	23	24	25	26	27	28	29	30	31	32	33	34
Top diagnosis	1	2	2	1	1	2	1	1	4	1	2	2	1	1	4	2	2	1	2	2	1	2	4	2	2	1	1	1	1	2	1	5	2	3
**Present symptoms entered and their entry order**																																		
Pain in more than one joint	4	6	5	1	1	3	5	5	2	6	5	5	2	1	1	6	4	6	6	4	5	x	4	7	5	3	3	7	2	8	1	x	5	4
Morning stiffness	1	3	2	3	4	4	x	4	4	4	3	4	1	3	3	4	2	4	4	3	3	6	2	6	4	2	2	5	3	6	3	3	4	3
Hand pain	x	x	9	x	x	x	1	2	x	x	1	3	x	5	5	1	x	1	x	x	x	x	x	x	x	x	6	1	x	1	x	x	x	x
Reduced flexibility in one´s fingers	x	1	x	5	2	2	2	1	x	1	2	x	5	x	x	2	x	3	2	x	1	3	x	3	x	1	x	x	1	2	x	1	x	x
Joint pain in fingers	x	5	1	4	5	x	4	x	x	x	4	x	4	x	4	5	3	5	5	1	4	2	5	1	6	5	x	6	4	7	5	x	7	5
Wrist pain	2	x	x	x	x	1	x	x	x	2	x	x	x	x	x	x	x	x	x	x	x	1	1	2	1	x	1	x	x	x	x	x	2	2
Reduced flexibility in the wrists	x	x	4	x	x	x	x	x	x	x	x	1	x	x	x	x	x	x	x	x	x	4	x	4	x	x	x	2	x	3	x	x	x	x
Reduced flexibility in multiple joints	5	x	3	x	x	x	x	x	1	x	x	x	x	x	x	x	x	x	x	x	x	x	x	x	x	x	4	x	x	x	4	x	x	x
Fatigue	3	2	x	2	3	x	3	3	3	3	x	2	3	2	2	3	6	2	3	2	2	5	3	5	3	4	5	3	5	5	2	2	3	x
Painful finger joints when pressure applied	6	4	6	x	6	x	6	x	x	5	x	x	x	x	x	x	5	x	x	5	x	x	x	x	x	x	7	x	x	9	x	4	6	x
Finger pain	x	x	x	x	x	x	x	x	x	x	x	x	x	x	x	x	x	x	1	x	x	x	x	x	2	x	x	4	x	4	x	x	1	1
Swollen finger joint	x	x	7	x	7	x	x	x	x	7	x	x	x	x	x	x	x	x	x	6	x	x	x	x	x	x	x	x	x	x	x	x	x	x
Recurring respiratory tract infection	x	x	8	x	x	x	x	x	x	x	x	x	x	x	x	x	x	x	x	x	x	x	x	x	x	x	x	x	x	x	x	x	x	x
General muscle pain	x	x	x	x	x	x	x	x	x	x	x	x	x	4	x	x	x	x	x	x	x	x	x	x	x	x	x	x	x	x	x	x	x	x
Arm pain	x	x	x	x	x	x	x	x	x	x	x	x	x	x	x	x	1	x	x	x	x	x	x	x	x	x	x	x	x	x	x	x	x	x

Top diagnoses 1: Rheumatoid arthritis, 2: Felty´s syndrome, 3: Osteoarthritis, 4: Fibromyalgia, 5: Snapping finger
